# NanoskinⓇ Bacterial Cellulose as a Scaffold for De-epithelialized Corneal Surface Healing

**DOI:** 10.18502/jovr.v21.18451

**Published:** 2026-10-06

**Authors:** Magda Viveiros, Cláudia Rainho, Natália Avelino, Carolina Gorgulho, Ramon Kaneno, Alexandre Fabro, Márcio Viveiros, Pierre Basmaji, Nick Di Girolamo, Silvana Schellini

**Affiliations:** ^1^Department of Ophthalmology, Medical School, São Paulo State University (Unesp), Botucatu, São Paulo, Brazil; ^2^Department of Chemical and Biological Sciences, Institute of Biosciences, São Paulo State University (Unesp), Botucatu, São Paulo, Brazil; ^3^Department of Pathology, University of Sao Paulo - USP, Ribeirao Preto, SP, Brazil; ^4^Department of Orthopedics and Traumatology, Medical School, São Paulo State University (Unesp), Botucatu, São Paulo, Brazil; ^5^Innovatecs Biotechnological Research and Development, São Carlos, SP, Brazil; ^6^Department of Ophthalmology, University of New South Wales, Sydney, Australia

**Keywords:** Bacterial Cellulose, Cornea, Healing, Nanoskin, Scaffold

## Abstract

**Purpose:**

This study investigates the ability of Nanoskin, a bacterial cellulose (BC) biofilm, to transfer human corneal epithelial cells (CECs) onto wounded rabbit corneas, evaluating integration and re-epithelialization of the ocular surface.

**Methods:**

Primary cultures of human mesenchymal stem cells (MSCs) were established and differentiated into CECs, which were then seeded onto Nanoskin BC or soft contact lenses. Wounded rabbit corneas were covered with human CECs on BC biofilms or contact lenses and maintained in organ culture for 5 days. The control group was left untreated. Integration and re-epithelialization were assessed using immunofluorescence (IF) staining with human anti-nuclei antibodies.

**Results:**

Wounded rabbit corneas successfully re-epithelialized with both scaffolds. IF staining confirmed the presence of human epithelial cells in the regenerating rabbit corneal epithelium for both Nanoskin BC and contact lens groups, confirming effective cell transfer. Morphometric analysis revealed that control wounded corneas had significantly fewer epithelial cells (*P <* 0.001) compared to those treated with cell transfer.

**Conclusion:**

Our findings demonstrate the feasibility of using Nanoskin BC as a scaffold for corneal cell transplantation, highlighting its potential as a promising biomaterial for treating ocular surface diseases. Future investigations are required to evaluate the transfer efficiency of MSCs, given their superior proliferative capacity and secretion of growth factors.

##  Introduction

Corneal surface diseases resulting from accidental mechanical, chemical, or thermal injuries are a common cause of vision loss. Severe damage to limbal stem cells can lead to persistent corneal epithelial defects,^[1]^ which are typically managed through donor tissue transplantation. However, this approach has several drawbacks, including the risk of rejection, limited availability of donor tissue, and variability in corneal graft survival. Overall, the estimated 10-year graft survival was 65%.^[2]^

As a result, scientific interest has shifted toward regenerative medicine, which aims to support host tissue regeneration by providing an appropriate microenvironment—including growth factors, cells, and/or signals^[3]^—as an alternative to conventional keratoplasty. Cells can be delivered to the corneal surface using scaffolds. The human amniotic membrane (HAM) is the most commonly used scaffold for delivering cells or tissues in ocular surface reconstruction. However, HAM has several limitations such as limited availability, the need for microbial screening, and its inherently semi-opaque nature.^[4]^

Nanocellulose is a linear polymer derived from cellulose with dimensions up to 100 nm and is produced by plants, bacteria, algae, and some marine invertebrates.^[5]^ The most relevant sources of nanocellulose for tissue engineering applications are bacteria from the genera *Komagataeibacter, Acetobacter,* and *Gluconacetobacter*.^[5,6,7]^ These microorganisms produce extracellular cellulose to form biofilms of varying thickness, which help maintain high oxygenation levels near the colony surface. Bacterial cellulose (BC) biofilms act as a protective barrier against desiccation, predatory bacteria, environmental stress, and UV radiation.^[7]^ These intrinsic properties make BC an ideal scaffold for protecting injured tissues, burn wound dressings, tissue regeneration, and temporary skin substitution.^[5,6,7]^

BC nanofiber scaffolds are a promising material for the adhesion, expansion, and transfer of various cell types, including mesenchymal stem cells (MSCs), corneal epithelial cells (CECs), and limbal epithelial stem cells.^[8]^ The surface of BC nanofibers can mimic the structure of extracellular matrix proteins, supporting the growth of corneal stroma,^[9]^ epithelial cells,^[10]^ and retinal pigment epithelium cells.^[11]^ In addition to their cell-carrying capabilities, BC scaffolds can be loaded with pharmacologically active substances such as epidermal growth factor (EGF), lidocaine, diclofenac, ceftriaxone, and fluconazole. These agents can enhance cell proliferation, reduce pain and local inflammation, provide antibacterial or antifungal effects, and be programmed for controlled absorption.^[6]^

The present study investigates the Nanoskin BC biofilm, evaluating its feasibility as a scaffold for cell transplantation in wounded rabbit corneas.

##  Methods

The study protocol was approved by the Research Ethics Committee of the Institution (ERB #1.376.415) and was conducted in accordance with the principles of the Association for Research in Vision and Ophthalmology (ARVO) Statement for the Use of Animals in Ophthalmic and Vision Research and the tenets of the Declaration of Helsinki. Informed consent was obtained from all human donors. No live animals were used throughout the study, and all experiments were performed in an *ex vivo* corneal setting.

### Nanoskin BC Production

Nanoskin is a BC biofilm produced by Innovatecs Biotechnological Products, Ltd (São Carlos, São Paulo, Brazil). It is derived from *Acetobacter xylinum* (ATCC 23769), a gram-negative, non-pathogenic, aerobic bacterium capable of synthesizing high levels of polysaccharides using only carbon and nitrogen sources.^[12]^ Fermentation was carried out in a bioreactor, yielding a culture medium with a pure 3D structure composed of an ultra-fine network of cellulose nanofibers ranging from 3 to 8 nm. The acetic fermentation process utilized glucose as a carbohydrate source and green tea as a nitrogen source, supplemented with chondroitin 4-sulfate (1% w/w) and hyaluronic acid (1% w/w) prior to bacterial inoculation. The culture medium remained unchanged throughout the production process, resulting in vinegar and a nanobiocellulose biomass.

### Primary Cultures of Mesenchymal Stem Cells

Human bone marrow samples were obtained from five patients aged 46 to 65 years during orthopedic humeral osteosynthesis procedures. The exclusion criteria included open fractures or infections. MSCs were isolated following the protocol described by Ramakrishnan et al (2013).^[13]^ Briefly, BM was diluted in three volumes of Dulbecco's Modified Eagle's Medium/Nutrient Mixture F-12 (DMEM/F12; Gibco, Grand Island, NY, USA). Six milliliters of diluted BM was layered over 3 mL of Ficoll-Paque Plus (GE Healthcare Life Science, Freiburg, Germany) and centrifuged at 1600 RPM 400 g for 30 min. The white cloudy interface between the Ficoll and medium was collected, transferred to a 15 mL tube, suspended in DMEM/F12 nutrient medium, and washed twice. Cells were seeded in two 25 cm^2^ culture flasks and incubated at 37°C with 5% CO_2_ in mesenchymal stem cell basal medium (MSCBM-CD, Lonza, Walkersville, MD, USA) supplemented with 20% fetal calf serum (FCS; Gibco, Grand Island, NY, USA). After 48 hours, non-adherent cells were removed. The medium was refreshed every 4 days until cells reached 80-90% confluence, at which point they were detached with 0.25% trypsin-EDTA (Gibco, Grand Island, NY, USA) and reseeded at a density of 500 cells/cm^2^ in 75 cm^2^ culture flasks until the third passage.

### Morphological Analysis and Cell Proliferation on BC Scaffolds

MSCs were seeded at a density of $2\times 10^{4}$ cells/cm^2^ onto Nanoskin BC and incubated at 37°C with 5% CO_2_ in MSCGM medium supplemented with 10% FCS for 6 hours, and for 1, 3, 7, 10 and 14 days. At each time point, samples were fixed in 2.5% glutaraldehyde for 4 hours at 4°C, washed in distilled water, and post-fixed in 0.5% osmium tetroxide for 30 minutes. Samples were then dehydrated through a graded ethanol series (7.5%, 15%, 30%, 50%, 70%, 90% and 100%), mounted on stubs, and sputter-coated with gold. Scanning electron microscopy (SEM; EVO LS 15, Carl Zeiss, Oberkochen, Germany) was used to assess MSC adhesion to the biomaterial.

### MSC Phenotyping

Human MSCs are characterized by their plastic adherence under standard culture conditions and their ability to differentiate into adipocytes, osteoblasts, and chondrocytes. At least 95% of the MSC population must express CD73, CD90, and CD105, while < 2% should express CD34, CD45, and CD14.^[14]^ Third-passage cells were phenotypically characterized by flow cytometry using the BD Stemflow hMSC Analysis Kit (BD Pharmingen, Sparks, MD, USA). Briefly, cells were detached with 0.25% trypsin, adjusted to a concentration of 5-10 $\times 10^{6}$ cells/mL, and incubated with monoclonal antibodies or isotypic controls [Supplementary Table 1] for 30 minutes at 2° to -8 °C, protected from light. After two washes with cold PBS containing 10% FBS, cells were resuspended in 250 μ L of the same buffer and analyzed using a FACS Canto II flow cytometer (BD Biosciences, San Jose, CA, USA) and Flow Jo software (Tree Star, Ashland, OR, USA).

### *In vitro* Differentiation of MSC

For osteogenic differentiation, third-passage MSCs ( $3.1\times 10^{3}$ cells/cm^2^) were seeded on a 10 mm round coverslip in 24-well plates with MSCBM medium (Lonza, Walkersville, MD, USA). After 24 hours of incubation at 37°C and 5% CO_2_, four wells were maintained in MSCBM as controls, while four others were treated with hMSC Osteogenic Differentiation Medium (Lonza, Walkersville, USA), containing DMEM supplemented with dexamethasone, glycerolphosphate, ascorbic acid, sodium pyruvate, proline, L-glutamine, gentamicin, amphotericin and ITS (insulin, transferrin, and selenium). The medium was refreshed every 3 days for 24 days. Calcium deposition was assessed by Alizarin Red S (Sigma-Aldrich, St. Louis, MO, USA). Cells were fixed with 4% paraformaldehyde (PFA), rinsed with ultrapure water, stained for 5 minutes, and washed three times before microscopy analysis. For adipogenic differentiation, third-passage MSCs ( $2.1\times 10^{4}$ /cm^2^) were seeded on coverslips in 24-well plates and cultured in MSCGM medium for 7 days until confluence. Control wells remained in MSCGM, while adipogenesis was induced using hMSC Adipogenic Differentiation Medium (Lonza, Walkersville, USA), alternating every 3 days with hMSC Adipogenic Maintenance Medium (Lonza, Walkersville, USA). Lipid accumulation was confirmed by Oil Red O staining (Sigma-Aldrich, St. Louis, MO, USA). Briefly, cells were fixed with 4% PFA, washed with PBS, stained for 2 hours, rinsed, and analyzed by microscopy.

### *In vitro* Differentiation of MSC into Corneal Epithelial Cells (CECs)

MSCs were differentiated into CECs using conditioned medium derived from human corneal epithelial cultures. Donor corneas unsuitable for transplantation due to low endothelial cell counts were used. Limbal and endothelial tissues were removed, and corneas were cut in 1×1 mm fragments and cultured in Dulbecco's Modified Eagle Medium and Ham's F-12 (DMEM/F12; Gibco, Grand Island, NY, USA) supplemented with 2.5 ng/mL EGF (Gibco, Frederick, MD, USA), 5 ng/mL ITS (insulin, transferrin, and selenium), 0.5 ng/mL cholera toxin, 50 μ g/mL hydrocortisone, 0.5% DMSO, 100 IU/mL penicillin G, 40 μ g/mL gentamicin, 2 μ g/ml amphotericin B, and 5% FCS (Sigma-Aldrich, Freiburg, Germany). Medium was changed every 3 days, and conditioned medium was collected until the third passage, filtered through a 0.2 μ m filter, and stored at 4° to 8°C. For differentiation, second-passage MSCs ( $3\times 10^{5}$ ) were seeded in 25 cm^2^ flasks with 10% FCS and 40% conditioned medium. Cultures were maintained at 37°C with 7% CO_2_, with medium changes every 3-4 days for 21 days. Differentiation was confirmed by flow cytometry using antibodies against pan-cytokeratin (IRG Bioscience, Rockville, MD, USA), CK3, and CK12 (Novus Biologicals, Littleton, CO, USA) [Supplementary Table 2]. Triplicate analysis was performed on five cultures, with MSCs cultured in DMEM/F12 serving as negative controls.

### *Ex vivo* Study of the Rabbit Corneas Re-epithelialization

CECs ( $∼1.25\times 10^{5}$ ), derived from MSCs, were seeded onto four round Nanoskin BC membranes (14 mm diameter) and four soft contact lenses (Focus Night & Day^®^; Lotrafilcon A, Ciba Vision, Duluth, GA, USA). These scaffolds were maintained in culture for 12 days in maintenance medium supplemented with 10% FCS and 20% conditioned medium from CECs. Ten whole eyes from euthanized healthy rabbits were aseptically enucleated within 2 hours postmortem, in accordance with the principles of the ARVO Statement for the Use of Animals in Ophthalmic and Vision Research. Eyelids were kept closed until enucleation. Under a laminar flow hood, a 5 mm diameter filter paper disc soaked in 1 N NaOH was applied to the central cornea for 60 seconds, followed by extensive rinsing with 0.9% saline for 10 minutes. Residual debris was removed using sterile cotton swabs. The cornea and limbus were excised from each globe, along with the iris and adherent ciliary body. Corneas were placed on sterile rubber bases with convex surfaces in 12-well plates to preserve their dome surface and prevent deformation under the weight of BC biofilm. De-epithelized corneas were then covered with either BC biofilms or contact lenses, with the seeded epithelial cells facing the corneal surface. Nutrient medium was added to fully immerse the samples and scaffolds. Two additional corneas served as untreated controls—wounded but not scaffolded—for the immunofluorescence (IF) analysis. Corneas were incubated at 37°C and 7% CO_2_ for 5 days, then subjected to IF staining using a human anti-nucleus antibody (Abcam, Cambridge, MA, USA) to identify human epithelial cells transferred to the rabbit corneas by the scaffolds. A previous study demonstrated that rabbit corneal alkali wounds created using this same method fully re-epithelialized within 5 days.^[15]^ Therefore, corneas were analyzed at these same time points to ensure complete epithelialization.

### Analysis of the Corneal Re-epithelialization

De-epithelialized rabbit corneas treated with the BC biofilms ( n=4 ) or contact lenses ( n=4 ) were cryosectioned and mounted on coverslips using optimal cutting temperature (OCT) compound. Sections were rinsed four times with PBS to remove OCT, fixed in acetone for 10 min at 2-8°C, and rinsed three additional times with PBS. A hydrophobic barrier was drawn around the tissue using a hydrophobic pen. Samples were blocked with Triton 0.5% for 10 minutes, followed by 5% BSA/PBS for 20 minutes and incubated overnight at 2-8°C with a human anti-nuclei primary antibody (Rockland Immuno Chemicals Inc., Gilbertsville, PA, USA; 909-301-D99) diluted 1:5 in 5% BSA/PBS. Negative controls were processed without primary antibody. After incubation, tissues were rinsed three times with PBS and incubated for 1 hour at room temperature with an Alexa Fluor 647-conjugated anti-human secondary antibody (Abcam, Cambridge, MA, USA), including the negative controls. Samples were rinsed twice with PBS, then stained with 4',6-Diamidino-2-Phenylindole, Dihydrochloride (DAPI; Abcam, Cambridge, MA, USA; ab228549) diluted 1:1000 in 5% BSA/PBS for 5 minutes [Supplementary Table 3]. After a final rinse, slides were mounted with anti-fade mounting media (Invitrogen, Carlsbad, CA, USA). Uninjured humans and rabbit corneas were used as controls in the IF assay.

The number of cells covering the wounded corneal surface was quantified by morphometric analysis using a Weibel reticle overlaid on a computer screen. This grid of randomly distributed points allows stereological estimation of area fractions. For each cornea, standardized random fields were selected, and the number of points coinciding with human epithelial cells was counted relative to the total number of points in the reticle. The ratio of points on cells to total points was expressed as “Area Fraction (%),” representing the proportion of the corneal surface covered by human epithelial cells.

### Statistical Analysis

Statistical analysis was performed using one-way ANOVA and Bonferroni's multiple comparisons test, with a significance level set at 5%. Analyses were conducted using GraphPad Prism 8 software (GraphPad Software Inc., La Jolla, CA, USA).

##  Results

### MSC Primary Cultures

Phenotypic characterization by flow cytometry confirmed that the analyzed samples were consistent with MSC populations, based on cell size and granularity. The cells [Figure 1A] exhibited high expression of stem cell markers: CD90 (average of 83.5%), CD105 (average of 75.4%), and CD73 (average of 96.1%). The means and standard deviations of MSCs expressing specific markers are shown in Supplementary Figure 1. They also showed negativity or very low expression of CD34, CD45, CD11b, CD19, and HLA-DR (average of 1.54%), which are typically used to identify potential contaminants such as hematopoietic stem cells. These findings validated the successful establishment of a homogeneous culture of human bone marrow-derived MSCs.

### MSC Differentiation Assays

MSC induced to differentiate into adipocytes were stained with Oil Red O, revealing numerous lipid vesicles within the cytoplasm. In contrast, control cells displayed only the lipids present in plasma membranes [Figure 1B]. MSCs induced to differentiate into osteocytes were stained with Alizarin Red S, demonstrating mineralized bone matrix nodules with calcium deposits. Non-induced control cells retained their elongated mesenchymal cell morphology [Figure 1C].

### MSC Growth on BC Biofilm

SEM revealed MSC adhesion to the surface of Nanoskin BC membranes only after 10 days of incubation [Figure 1D, arrows]. Prior to this period, only debris and structural irregularities of the BC membrane were observed.

### MSC Differentiation into Corneal Epithelial Cells (CECs) *In Vitro*

Flow cytometry analysis confirmed that MSCs differentiated into CECs, as evidenced by the expression of cornea-specific cytokeratin markers: CK3 (95.3% of positive cells vs 38% in controls); CK12 (93.4% vs 42.5% for controls), and pan-cytokeratin (95.3% vs 19.4% for control). These results validated the effectiveness of the cell differentiation protocol [Figure 2A].

**Figure 1:**
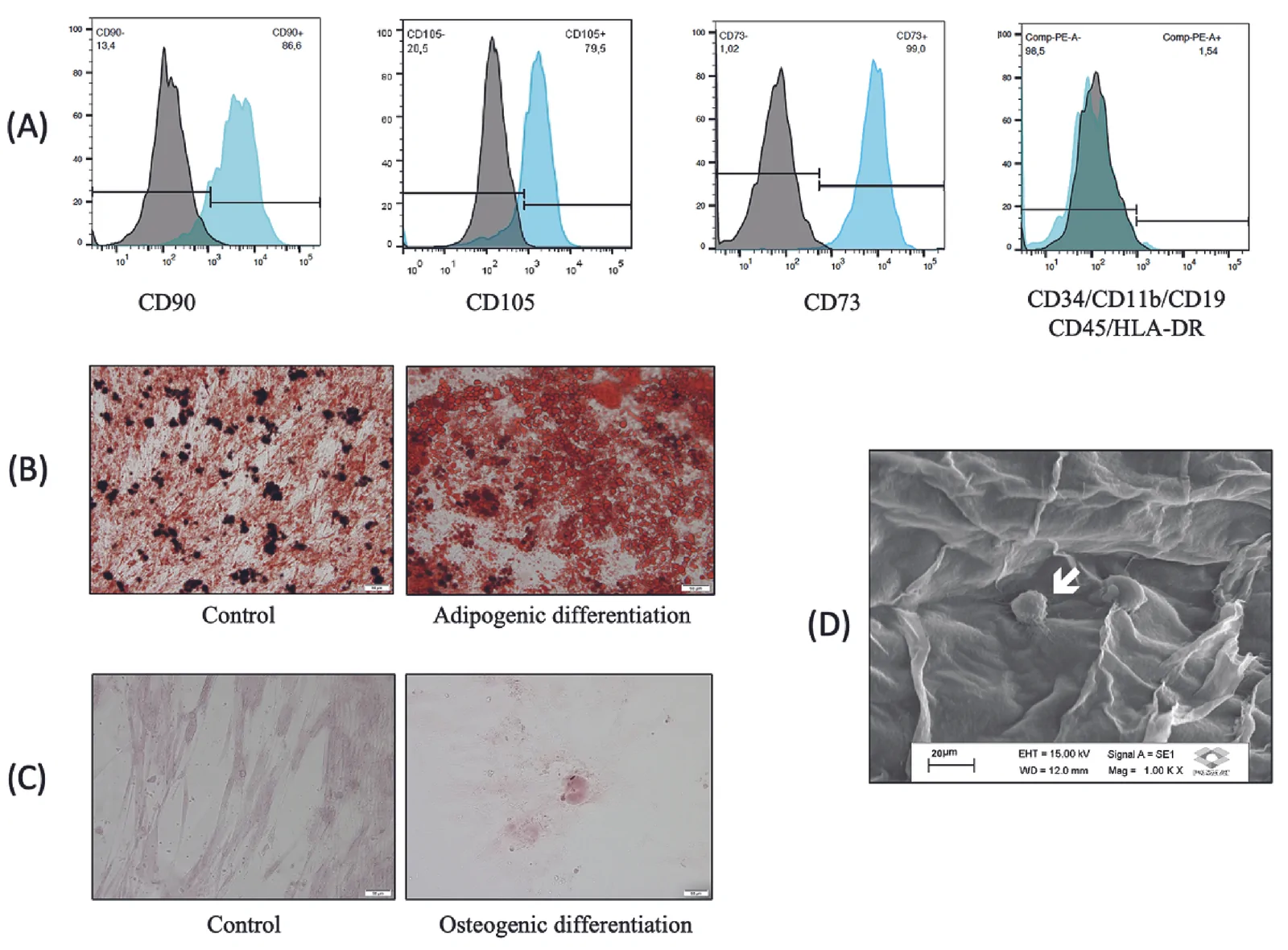
Characterization of MSC. (A) MSCs were positive for CD90, CD105, and CD73 and negative for CD34, CD11b, CD19, CD45, and HLA-DR as analyzed by flow cytometry. (B)MSCs treated with adipocytic inductive medium (Adipogenic differentiation) showed lipid vesicles in the cytoplasm stained by Oil Red O and the controls stained only the lipid present in the plasmatic membranes of the cells. Scale bar = 50 μ m. (C) MSCs cultured with osteogenic-inductive medium stained with Alizarin Red S, showing the formation of bone matrix mineralization nodules, with calcium deposits (osteogenic differentiation). Scale bar = 50 μ m. (D) Scanning electron microscopy (SEM) of Nanoskin BC with MSC adhered to its surface after 14 days of incubation (arrow). Scale bar = 20 μm.

**Figure 2:**
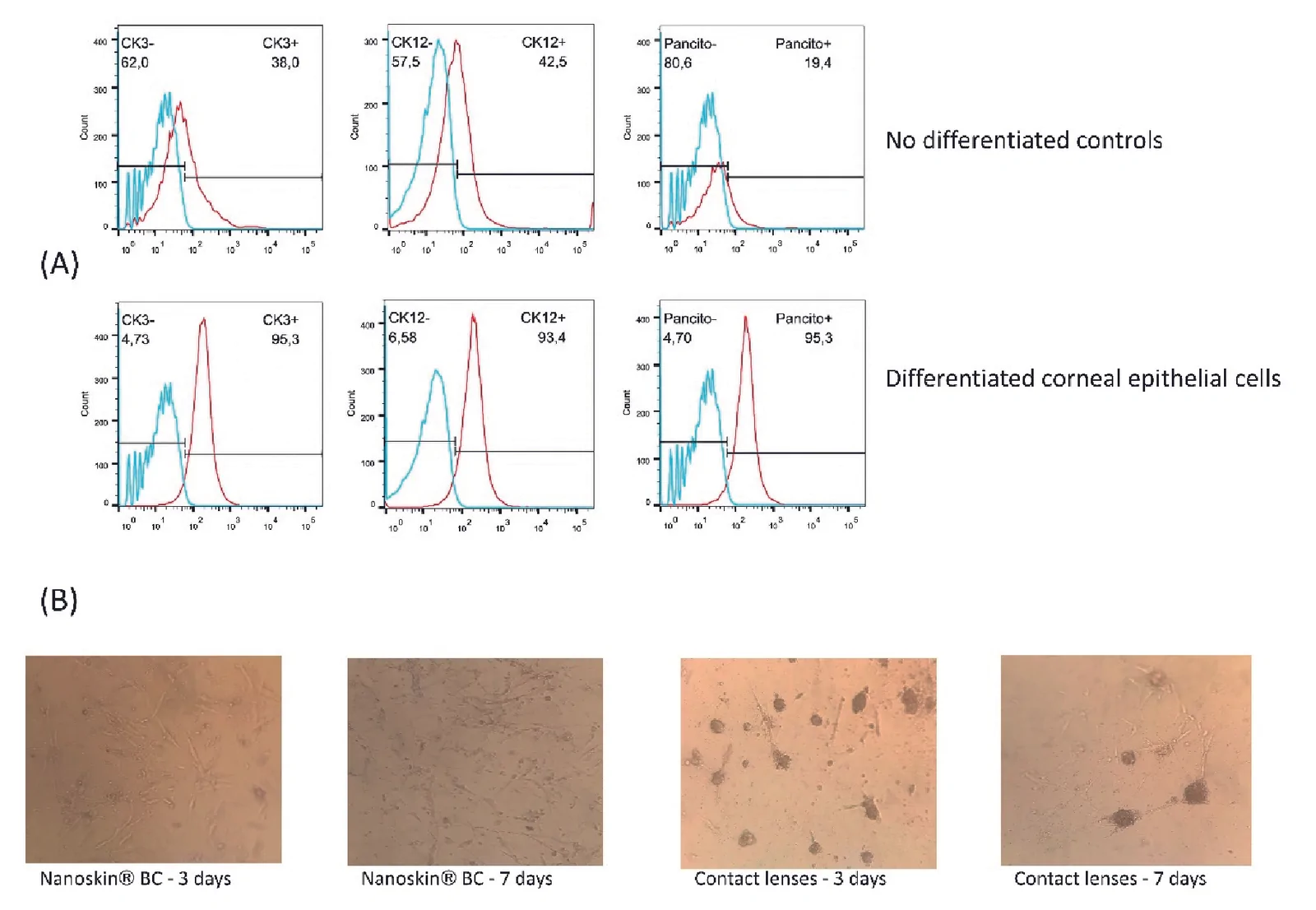
(A) Flow cytometry analysis of MSCs induced to differentiate into corneal epithelial cells showed the labeling for the cornea-specific markers CK3 and CK12. The non-differentiated controls were labeled in 38% for CK3 and 42.5% for CK12. The differentiation-induced group was labeled in 95.3% for CK3 and 93.4% for CK12. Pancytokeratin marker labeled 95.3% of the induced group and 19.4% of the control group. (B) Corneal epithelial cells proliferation on Nanoskin BC and on soft contact lenses.

### BC Scaffold for Corneal Re-epithelialization

CECs seeded on Nanoskin BC biofilms exhibited higher proliferation rates compared to those seeded on soft contact lenses [Figure 2B]. Wounded rabbit corneas were successfully epithelialized using both scaffolds, as demonstrated by DAPI staining, which revealed cellular coverage across the entire surface. IF staining with human anti-nucleus antibody confirmed the presence of human epithelial cells in the rabbit corneal epithelium, indicating successful cell transfer and wound regeneration by both Nanoskin BC [Figures 3A–C] as well as soft contact lenses [Figure 3D–F].

Control wounded corneas showed significantly fewer epithelial cells in the IF morphometric analysis ( P<0.001 ) [Figure 4] and lacked human cells in the epithelial surface [Figures 3G–I]. Treated corneas exhibited multiple epithelial cell layers across the entire surface, whereas untreated controls displayed increased stromal thickening, as evidenced by the elevated number of stromal cells observed by DAPI staining [Figures 3H & I]. Images of the negative controls from the IF assay, processed without the primary antibody, demonstrated only DAPI labeling, and are provided in the supplementary data [Supplementary Figure 2].

##  Discussion

The present study aimed to evaluate the potential of Nanoskin BC as a scaffold for cell transplantation in wounded rabbit corneas using both *in vitro* and *ex vivo* approaches. Our findings demonstrate that Nanoskin BC is a promising scaffold for transferring CECs to injured corneal surfaces. Its three-dimensional structure provides a favorable environment for cell growth, enhancing surface area for adhesion and proliferation, which facilitated successful cell transfer and re-epithelialization, as confirmed by the presence of human cells in the rabbit corneal epithelium.

Nanoskin BC supported the proliferation of human CECs, consistent with previous studies involving rabbit CECs.^[9]^ Multiple studies have highlighted BC's potential as a cell carrier for transplantation due to its ability to support mammalian cell attachment and proliferation.^[16,17,18]^ Its interconnected fibrillar nanostructure offers excellent intrinsic properties, making it widely applicable in tissue engineering.^[19]^ BC is also being explored as a biomaterial for artificial corneas.^[9,20,21]^ Its hydrophilicity, flexibility, and mechanical stability make it a robust scaffold for corneal regeneration.^[22]^ Additionally, BC's conformability allows it to be shaped like a contact lens, protecting seeded cells during transfer.^[19]^ Its biocompatibility, biodegradability, and ability to mimic extracellular matrix collagen fibrils also make BC suitable as a bioink for 3D printing applications.^[23]^

SEM analysis revealed MSC proliferation on Nanoskin BC surfaces after 10 days of culture, consistent with other studies.^[24]^ The nanofiber structure increased the available surface area for cell adhesion [Figure 1D], enhancing cell density on the scaffold. In addition, CECs seeded on BC biofilms showed greater proliferation than those on soft contact lenses [Figure 2B], probably due to the hydrophobic nature of the lenses hindering cell adhesion. Studies on biofunctionalized BC scaffolds have shown improved growth and differentiation of human pluripotent stem cells into dopaminergic neuronal progenitors.^[16]^ Recent modifications to BC surfaces, such as drying and plasma treatment, have further enhanced porosity and hydrophilicity, improving cell adhesion and growth.^[25]^

Previous research confirmed that Nanoskin BC did not induce immunogenic or cytotoxicity responses in human lymphocytes. Moreover, MSCs in contact with Nanoskin BC did not produce inflammatory cytokines IL-6, IL-8, and TNF- α ; but instead secreted elevated levels of IL-10, an anti-inflammatory cytokine that may reduce inflammation and neovascularization during the healing process.^[26]^ Beyond its inherent properties, BC can be functionalized with pharmacologically active substances to promote cell growth, provide antibacterial effects, or reduce local inflammation as part of a drug delivery system.^[16,19,21]^

**Figure 3:**
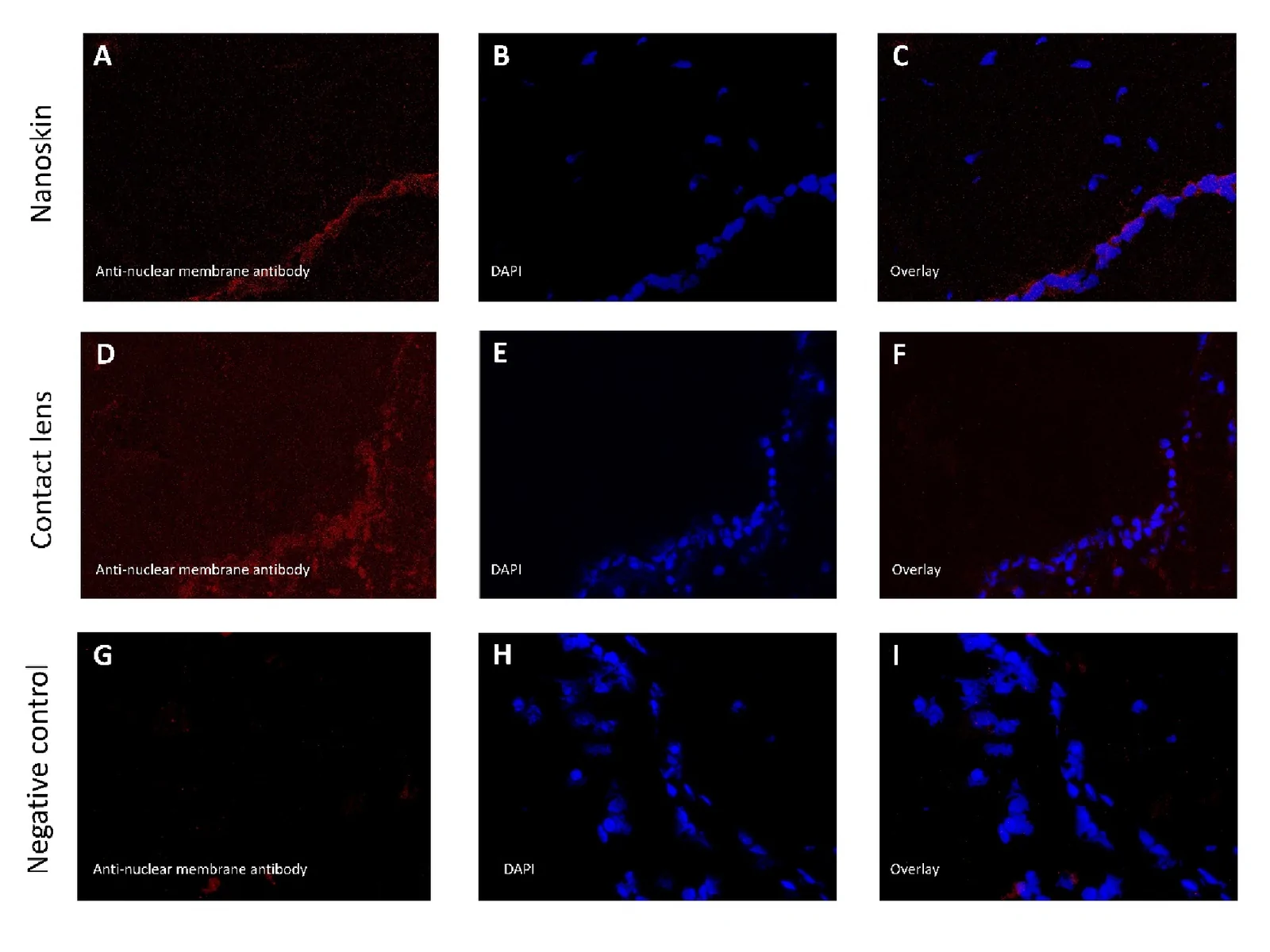
Immunofluorescence staining with human anti-nuclear membrane antibody of the de-epithelialized rabbit corneas using Nanoskin BC biofilms and soft contact lenses as a scaffold for the transfer of human corneal epithelial cells. The human cells have nuclear membranes labeled in red by the human anti-nuclear membrane antibody, surrounding the DAPI blue labeling of the DNA. (A–C) De-epithelialized rabbit cornea treated with Nanoskin BC as a scaffold for human epithelial cells transplantation. The DAPI labeling allows the exact localization of the cellular nuclei, which are also marked in their membranes by the human anti-nuclear membrane marker (double labeling).(D–F) De-epithelialized rabbit cornea treated with a soft contact lens as a scaffold for the transplantation of human epithelial cells. The human cells had the nuclear membranes labeled in red (Alexa 568) by a human anti-nuclear membrane marker. (G–I) Negative control of the study: de-epithelialized rabbit cornea without treatment with cell transplantation; there are no human cells in the corneal epithelium.

**Figure 4:**
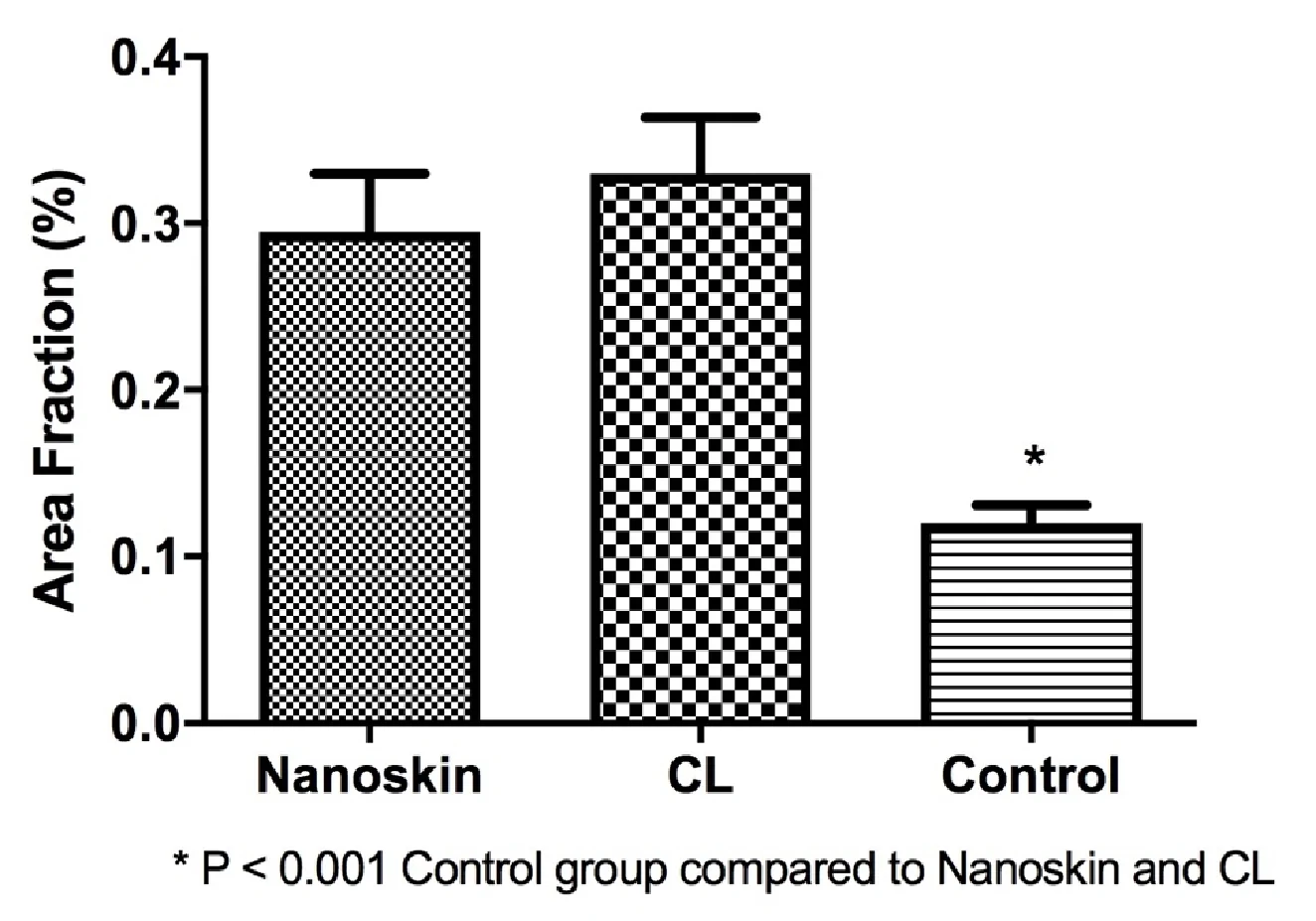
Morphometric study of the cells epithelializing the rabbit corneas. The untreated controls resulted in fewer cells than the Nanoskin and the contact lenses (CL) groups (*P < 0.001).

In the *ex vivo* study, IF staining with human anti-nucleus antibody confirmed the presence of human epithelial cells in the rabbit corneal epithelium, supporting wound regeneration with both scaffolds. Nanoskin BC demonstrated its ability to adhere and proliferate CECs, enabling their transfer to the wounded corneal surface. Treated corneas exhibited multiple epithelial layers, whereas untreated controls showed stromal thickening and increased stromal cell density, as revealed by DAPI staining, suggesting potential stromal scarring. The human cornea positive control confirmed the presence of human cells by showing red nuclear membrane labeling surrounding the blue DAPI-stained nuclei. Previous studies have shown that rabbit CECs cultured on BC scaffolds retain CK3 expression, indicating that BC does not interfere with corneal epithelium marker expression.^[10]^

Limitations of this study include the use of a mild to moderate central corneal de-epithelialization model that did not affect limbal stem cells, which are more challenging to regenerate. A longer observation period in the *ex vivo* study could provide insights into long-term stability. Additionally, the lack of quantitative data comparing CEC proliferation on BC and the absence of a control group with de-epithelialized corneas covered with scaffolds but without cells limit the interpretation of BC's intrinsic effects on epithelization.

The advantages of Nanoskin BC scaffolds over conventional contact lenses include enhanced cell adhesion facilitated by their three-dimensional nanofibrillar architecture, contrasting with the inherently hydrophobic surfaces of contact lenses. Furthermore, these scaffolds allow for the incorporation of pharmacologically active compounds.

Further studies should explore the potential of loading Nanoskin BC with pharmacologically active agents to enhance cell growth and modulate inflammation in corneal regeneration, and the evaluation of the transfer efficiency of MSCs, given their superior proliferative capacity and secretion of growth factors.

In summary, Nanoskin BC biofilm is an efficient scaffold for seeding and proliferating CECs, enabling their transfer to wounded corneal surfaces. It represents a promising alternative for treating de-epithelialized corneal injuries and advancing regenerative strategies in ophthalmology.

##  Financial Support and Sponsorship

This study was funded by the São Paulo State Research Foundation (Fundaç ao de Amparo à Pesquisa do Estado de São Paulo – FAPESP), under grant # 2015/10727-0, and by the UNESP Research Rectory, under grant #169.

##  Conflicts of Interest

None of the authors, except Pierre Basmaji, have financial interests or employment arrangements with any of the companies in this manuscript or with competing companies.
